# Sequential dual-targeting biomimetic nanovesicles for bone marrow–specific delivery of bortezomib in multiple myeloma

**DOI:** 10.3389/fbioe.2025.1714613

**Published:** 2025-11-17

**Authors:** Chongyan Lu, Zhifang Xiao, Hanqing Zhang, Peng Zhang, Yang Gao, Ling Ouyang, Xianjun He, Na Han, Jinfeng Zhang, Mengshan Guan, Yueqi Feng, Yonghua Li

**Affiliations:** 1 Guangzhou University of Chinese Medicine, Guangzhou, China; 2 Department of Hematology, General Hospital of Southern Theatre Command of PLA (People’s Liberation Army), Guangzhou, China; 3 The First School of Clinical Medicine, Southern Medical University, Guangzhou, China

**Keywords:** multiple myeloma, targeted drug delivery, nanoparticles, bortezomib, biomimetic nanocapsules, dual targeting

## Abstract

**Introduction:**

Inefficient bone marrow targeting remains a major barrier to improving clinical outcomes in multiple myeloma (MM). Although bortezomib (BTZ), a first-line proteasome inhibitor, exhibits potent antitumor activity, its short half-life, dose-limiting off-target toxicity, and pronounced neurotoxicity severely constrain therapeutic utility.

**Methods:**

Here, we report the design of a sequentially dual-targeted biomimetic nanoplatform that integrates the high affinity of alendronate for hydroxyapatite in the bone matrix with the homotypic targeting capability of MM cell membranes, thereby enabling a hierarchical “bone-first, tumor-next” delivery paradigm. This two-stage navigation strategy allows the nanoparticles to anchor specifically within the bone microenvironment and subsequently achieve precise MM cell recognition via membrane adhesion molecules, leading to enhanced intralesional BTZ accumulation and retention.

**Results:**

As a result, proteasome inhibition and apoptosis induction were markedly amplified. Both in vitro and in vivo studies demonstrated that this nanoplatform significantly prolonged survival in MM-bearing mice: all animals in the PBS control group succumbed within 29 days, whereas 100% of BTZ@PLGA/EM-treated mice survived to day 45 (p < 0.001), accompanied by reduced systemic toxicity.

**Discussion:**

Collectively, this work addresses a central challenge of drug delivery within the bone marrow niche and provides a promising strategy with strong translational potential for MM therapy.

## Introduction

Multiple myeloma (MM) is a lethal hematologic malignancy that accounts for approximately 10% of all blood cancers (Donk et al., 2021). It is defined by the clonal expansion of malignant plasma cells within the bone marrow, where they are shielded by the bone marrow microenvironment, thereby driving drug resistance and the emergence of relapsed/refractory disease (Hideshima et al., 2007). Despite substantial therapeutic progress, MM remains essentially incurable (Donk et al., 2021). The proteasome inhibitor bortezomib (BTZ), a frontline therapy, induces apoptosis by blocking proteasome activity (Manasanch and Orlowski, 2017; Whitby et al., 2000); however, its clinical utility is hindered by a short half-life, limited stability, nonspecific biodistribution, and dose-limiting neurotoxicity (Arastu-Kapur et al., 2011; Zajączkowska et al., 2019). These challenges underscore the pressing need for targeted strategies that can efficiently direct therapeutics to the bone marrow while minimizing systemic exposure.

Nanomedicine-based approaches have emerged as promising solutions to these limitations (Deshantri et al., 2018; De la Puente and Azab, 2017). Diverse formulations—including liposomes, polymers, and dendrimer-based systems—have been developed to improve pharmacokinetic behavior and mitigate the systemic toxicities of agents such as BTZ and doxorubicin (Hu et al., 2019). Ligand-modified nanoparticles (e.g., targeting CD38 or BCMA) have shown enhanced cellular uptake and preclinical efficacy (Yu et al., 2021; Larson et al., 2023), while liposomal BTZ formulations increase marrow accumulation and reduce neurotoxicity (Cao et al., 2023; Ozaki et al., 2007). Nevertheless, most current platforms rely on single targeting modalities, such as monovalent ligand–receptor interactions or passive accumulation (Chen et al., 2017; Qu et al., 2022; Guimarães et al., 2023), which are insufficient to achieve robust specificity and deep penetration within the highly complex bone marrow niche. More recently, biomimetic nanocarriers derived from cell membranes have attracted wide interest owing to their excellent biocompatibility and intrinsic targeting capacity (Swa et al., 2014; Detappe et al., 2023). In particular, their ability to recapitulate the “homing” behavior of source cells enhances accumulation within the bone marrow microenvironment (Chang et al., 2023; Gu et al., 2019). Nevertheless, natural cell membranes face several challenges, including limited targeting precision, rapid clearance by the immune system, and difficulties in coordinating multiple targeting signals (Lin et al., 2021). To address these issues, engineering strategies—such as the directed modulation of membrane protein composition or the integration of diverse targeting moieties—hold promise for substantially enhancing their dual-targeting capability toward both bone marrow tissue and tumor cells, thereby enabling more precise, efficient, and durable drug delivery (Nadar et al., 2017).

**FIGURE 1:**
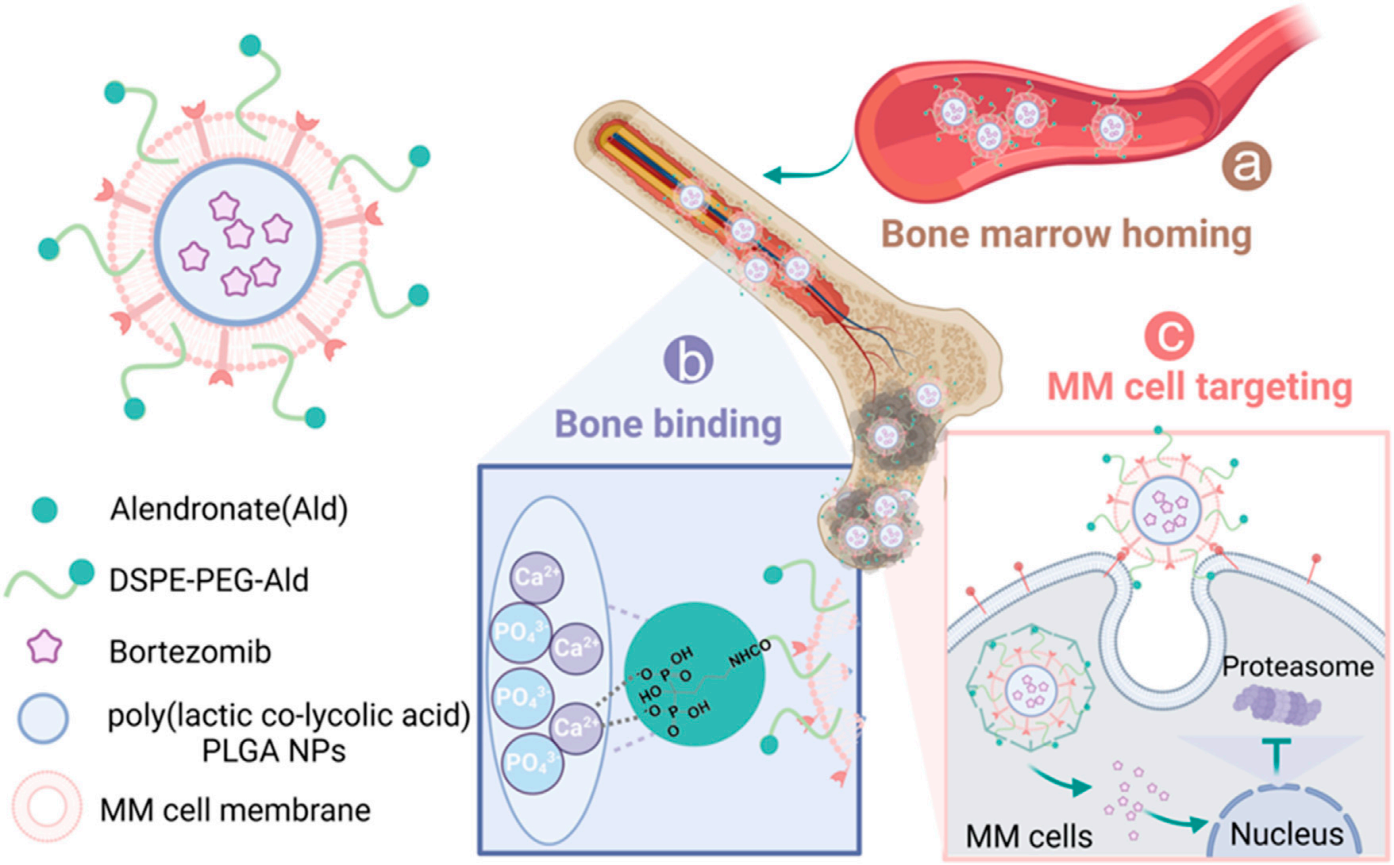
Schematic representation of sequential-targeted engineered cell membrane biomimetic nanovesicles for multiple myeloma therapy. **(a)** After intravenous injection, the nanocapsules BTZ@PLGA/EM enter the bone marrow from the blood circulation system due to the bone marrow homing ability of MM cells themselves; **(b)** Alendronate, as a Ca^2+^ ligand, mediated the preferential accumulation of nanovesicles in bone; **(c)** The MM cell membrane further mediates the targeted action of nanocapsules on MM tumor cells. After specific uptake of nanocapsules by MM cells, BTZ is released and acts on the proteasome to induce apoptosis of MM cells.

Although previous studies have attempted to improve drug delivery through single-targeting strategies (Yu et al., 2021; Larson et al., 2023), achieving cascade targeting within the complex bone marrow microenvironment remains a formidable challenge (Gu et al., 2019). To address this bottleneck, we herein propose and construct a sequential dual-targeting biomimetic nanoplatform designed to overcome the limitations of low delivery efficiency and high systemic toxicity in MM therapy. Specifically, we employed biodegradable poly(lactic-co-glycolic acid) (PLGA) as the core carrier to encapsulate the frontline drug bortezomib, and modified the surface with alendronate (Ald) to enable preferential anchoring to hydroxyapatite in the bone microenvironment, thereby achieving primary bone targeting (Sherr, 1996; Green, 2016). Building upon this, we further camouflaged the nanoparticles with MM cell membranes, which preserve adhesion molecules and endow secondary homotypic recognition as well as a ‘bone marrow homing’ property (Gu et al., 2019). Distinct from conventional strategies that rely solely on single ligands or single cell-membrane mimicry, our design integrates bone targeting and homotypic recognition into a unified sequential recognition system (Nadar et al., 2017). As illustrated in Figure 1, the nanovesicles are first localized at bone lesions via Ald mediation (Sherr, 1996; Green, 2016), subsequently bind to and are internalized by MM cells through membrane-derived molecules (Gu et al., 2019), and ultimately release BTZ to trigger tumor cell apoptosis (Cao et al., 2023). Both *in vitro* and *in vivo* experiments demonstrated that this nanoplatform, by virtue of its hierarchical targeting capability, markedly enhanced selective uptake by MM cells and drug accumulation at lesion sites (Swa et al., 2014; Nadar et al., 2017), effectively boosted the proteasome inhibitory activity and antitumor efficacy of BTZ (Cao et al., 2023), significantly prolonged the median survival of MM-bearing mice, and concurrently alleviated systemic toxicity and neurotoxicity. In summary, this study not only presents an efficient bone marrow–specific precision drug delivery strategy, but also provides an extensible avenue for developing nanotherapeutics against other hematological malignancies (Nadar et al., 2017).

## Results

### Characterization of BTZ@PLGA and BTZ@PLGA/EM nanovesicles

**FIGURE 2:**
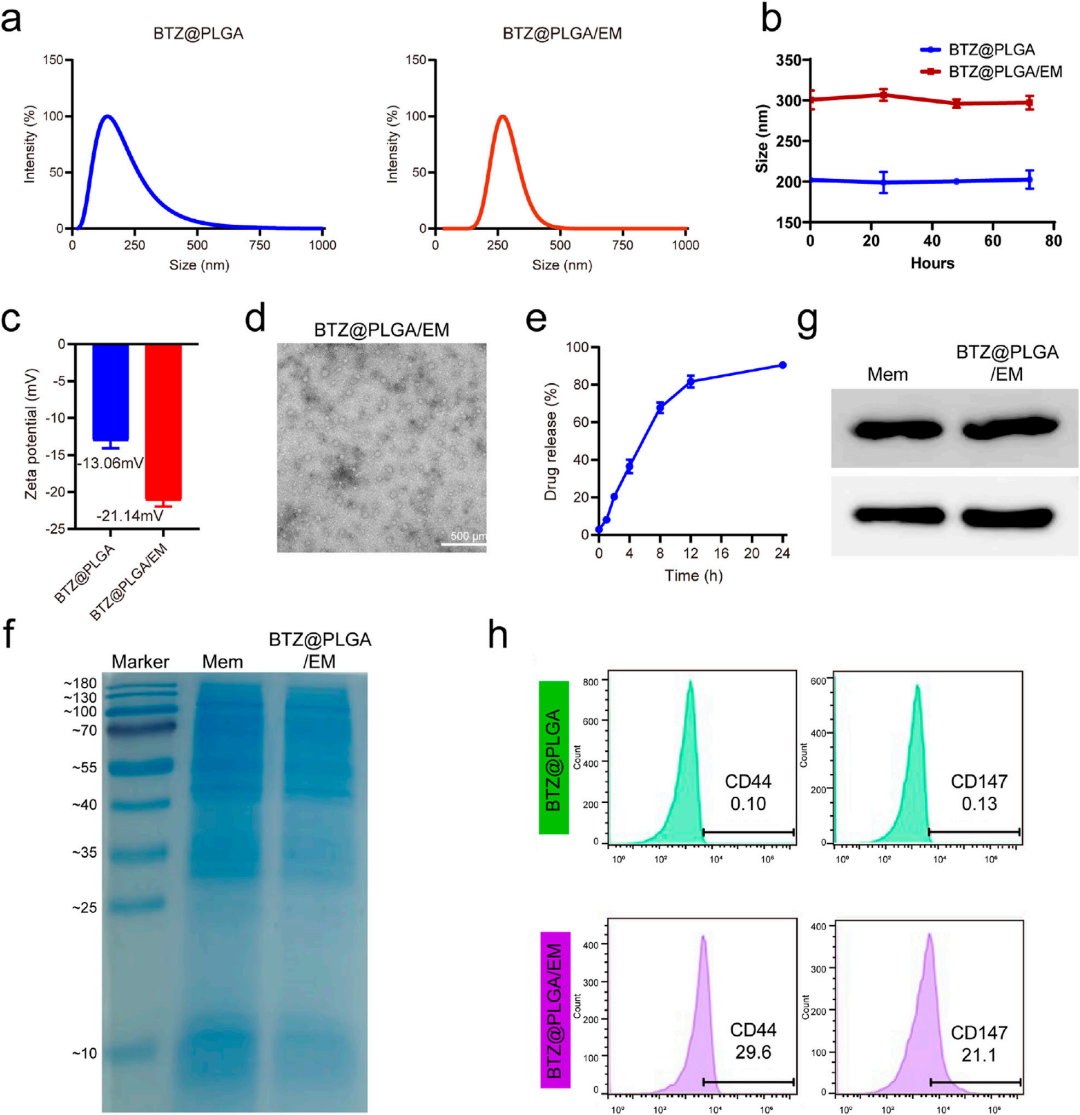
Representation of BTZ@PLGA/EM. **(a)** Particle size distributions of BTZ@PLGA nanoparticles and BTZ@PLGA/EM nanocapsules coated with engineered cell membranes. **(b)** Stability evaluation of BTZ@PLGA/EM nanocapsules by monitoring particle size at 0, 24, 48, and 72 h **(c)** The zeta potential of BTZ@PLGA and BTZ@PLGA/EM. **(d)** The TEM images of BTZ@PLGA/EM. **(e)** BTZ release curves of BTZ@PLGA/EM in PBS at different time points. **(f)** SDS-PAGE results of BTZ@PLGA/EM nanocapsules coated with MM membranes **(g)** Western blot was used to detect CD44 and CD147 on MM cell membranes and on BTZ@PLGA/EM nanocapsules coated with MM cell membranes. **(h)** Flow cytometry results of CD44 and CD147 on MM cell membranes and on BTZ@PLGA/EM nanovesicles coated with MM cell membranes.

Dynamic light scattering (DLS) analysis reconfirmed the particle size of BTZ@PLGA nanoparticles synthesized by the emulsification method as shown in Figure 2a, and the particle size was uniformly dispersed around 200 nm. The amount of BTZ encapsulated in PLGA reached 70% (Supplementary Figure S1). It has been reported that cancer cell membranes can be targeted to homologous tumor sites (Swa et al., 2014), and bisphosphonates are preferentially deposited in bone marrow after systemic administration, especially in sites with high bone turnover (Sherr, 1996). Therefore, in order to endow nanoparticles with specific targeting capabilities, cancer cell membranes and synthetic DSPE-PEG-Ald were combined with BTZ@PLGA nanoparticles by extrusion to produce BTZ@PLGA/EM nanovesicles. The particle size of these nanovesicles was uniformly distributed around 300 nm and larger than BTZ@PLGA (Figure 2a). The good stability of both BTZ@PLGA and BTZ@PLGA/EM nanocapsules was confirmed, as their particle sizes remained stable at approximately 200 nm and 300 nm, respectively, with no significant changes observed from 0 to 72 h (Figure 2b). The potential changed from −13.06 mV to −21.14 mV after encapsulation (Figure 2c), this indicates that the membrane was successfully coated on the nanoparticles. The transmission electron microscopy (TEM) images also proved the successful synthesis of nanovesicles (Figure 2d). The BTZ@PLGA/EM nanovesicles were stable and could release BTZ slowly, the release of BTZ was not complete until 24 h (Figure 2e). The particle size and potential of the nanovesicles did not change significantly after being exposed to PBS for 24 h. With the extension of time, the particle size gradually increased and the potential gradually decreased, which may be due to the gradual release of BTZ and the destruction of the structure of the nanovesicles (Supplementary Figure S2).

The results of sodium dodecyl sulfate polyacrylamide gel electrophoresis (SDS-PAGE) indicated that the nanovesicles retained membrane proteins (Figure 2f). Previous studies have shown that MM cells can mediate the “bone marrow homing” effect through the surface CD44 and CD147 molecules, Western blot analysis and flow cytometry showed that BTZ@PLGA/EM nanocapsules retained CD44 and CD147 proteins on the membrane of MM cells (Figures 2g,h; Supplementary Figure S3).

### Tumor cell targeting and In vitro antitumor activity of BTZ@PLGA/EM nanocapsules

**FIGURE 3:**
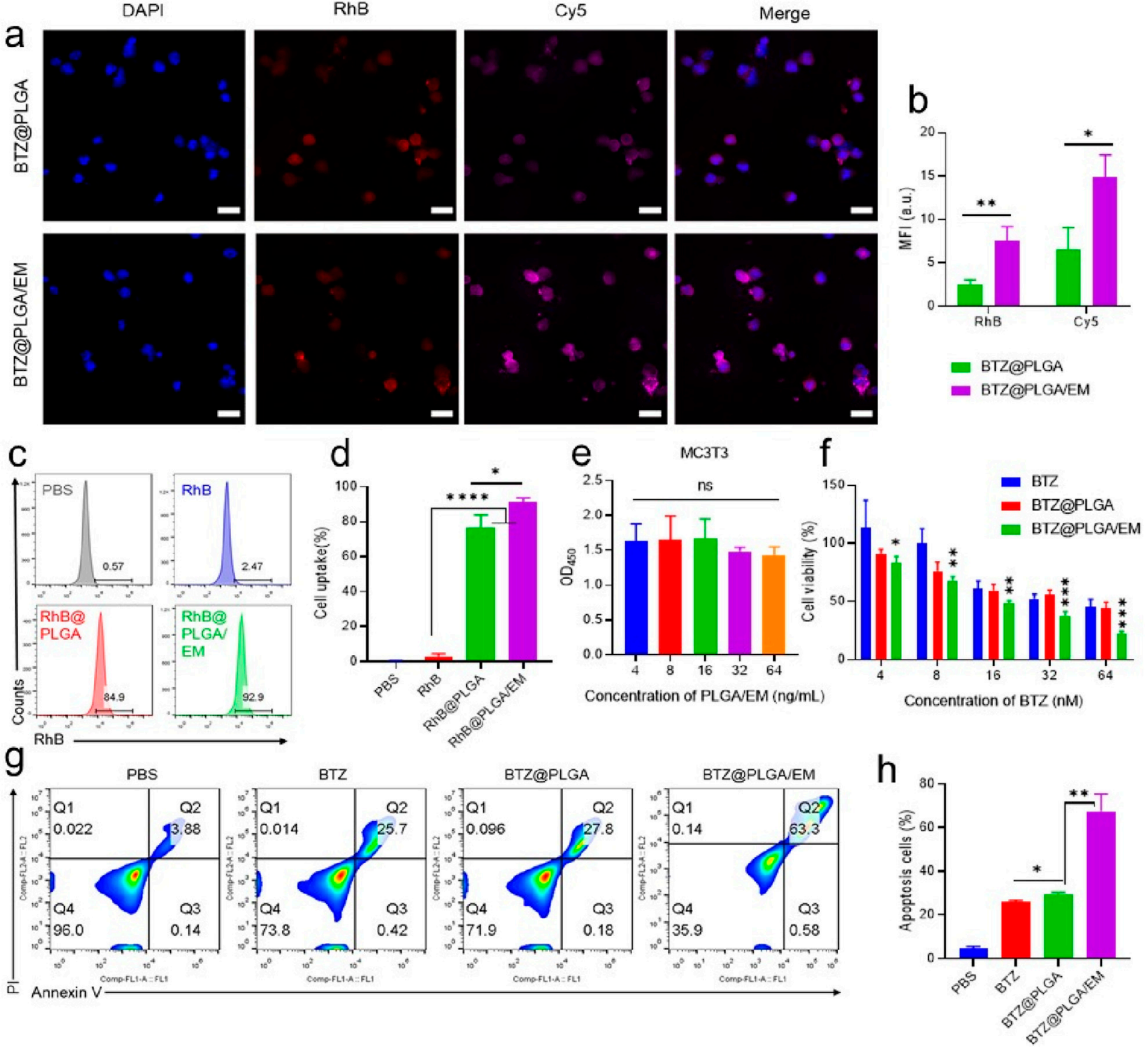
Endocytosis of BTZ@PLGA/EM by MM cells and killing effect of BTZ@PLGA/EM on MM cells. **(a)** The nucleus of MM was labeled with DAPI, and the BTZ@PLGA and BTZ@PLGA/EM nanocapsules loaded with RhB dye and Cy5 labeled with cell membrane were co-incubated with MM cells, respectively. The co-localization of nanocapsules and cells was observed by CLSM. **(b)** Fluorescence quantitative analysis of CLSM images. **(c)** Flow cytometry was used to detect the endocytosis of RhB-loaded nanocapsules by MM cells. **(d)** Quantification of cellular endocytosis by flow cytometry. **(e)** Toxicity assay of various concentrations of BTZ@PLGA/EM on MC3T3 cells. **(f)** Toxicity assays of different concentrations of BTZ, BTZ@PLGA and BTZ@PLGA/EM on MM cells. **(g)** Apoptosis/necrosis assay of MM cells after different treatments. **(h)** Quantitative analysis of apoptosis and necrosis. Data are shown as mean ± SD (n = 3). Statistical significance was analyzed by one-way ANOVA followed by Tukey’s test. p < 0.05, p < 0.01, p < 0.001.

To test whether EM would enhance the targeting effect of nanocapsules to cells *in vitro*. RhB dye was loaded into PLGA to track nanovesicles and EMs were labeled with Cy5 dye, myeloma cells were labeled with DAPI. After co-incubation of the nanomaterials with myeloma cells for 4 h, the co-localization of the materials and cells was observed by CLSM. It was observed that the fluorescence intensity of RhB and Cy5 dyes in cells incubated with BTZ@PLGA/EM was higher than that in cells incubated with BTZ@PLGA, indicating that cancer cell membranes can enhance the uptake of nanomaterials by cancer cells and have the effect of targeting cancer cells (Figure 3a). The same results were obtained for the quantitative data of the pictures (Figure 3b). Flow cytometry results after co-incubation of the materials with cells also showed that more BTZ@PLGA/EM nanovesicles were endocytosed in MM cells (Figures 3c,d).

It has been reported that the level and activity of proteasome in tumor tissues are significantly higher than those in normal tissues (Manasanch and Orlowski, 2017). Therefore, BTZ as a proteasome inhibitor should be less toxic to normal cells than to cancer cells. From the results of *in vitro* cytotoxicity experiments, it can be seen that BTZ@PLGA/EM has little toxicity to normal MC3T3 cells (Figure 3e), moreover, high concentrations of BTZ showed no significant toxicity to MC3T3 cells, while it has obvious cytotoxicity to MM cells, with the increase of concentration, the killing effect on MM cells was enhanced (Figure 3f). At the same time, BTZ and BTZ@PLGA were less toxic to MM cells than BTZ@PLGA/EM, which proved that EM had a targeting effect (Figure 3f). Live/dead staining experiments also demonstrated that BTZ@PLGA/EM killed more MM cells than BTZ, BTZ@PLGA (Supplementary Figure S4). Due to the inhibitory effect of BTZ on the proteasome, the intracellular stress pressure will increase and apoptosis will occur. Apoptosis of MM cells induced by BTZ@PLGA/EM was verified by labeling necrotic cells with PI and selectively binding of FITC labeled Annexin V to phosphatidylserine (PS) on the surface of apoptotic cells. The results showed that the cells treated with BTZ@PLGA/EM had more apoptosis and necrosis (63.3%) than those treated with PBS, BTZ and BTZ@PLGA (Figures 3g,h).

### Regulation of cell cycle and apoptosis-related proteins by BTZ@PLGA/EM

**FIGURE 4:**
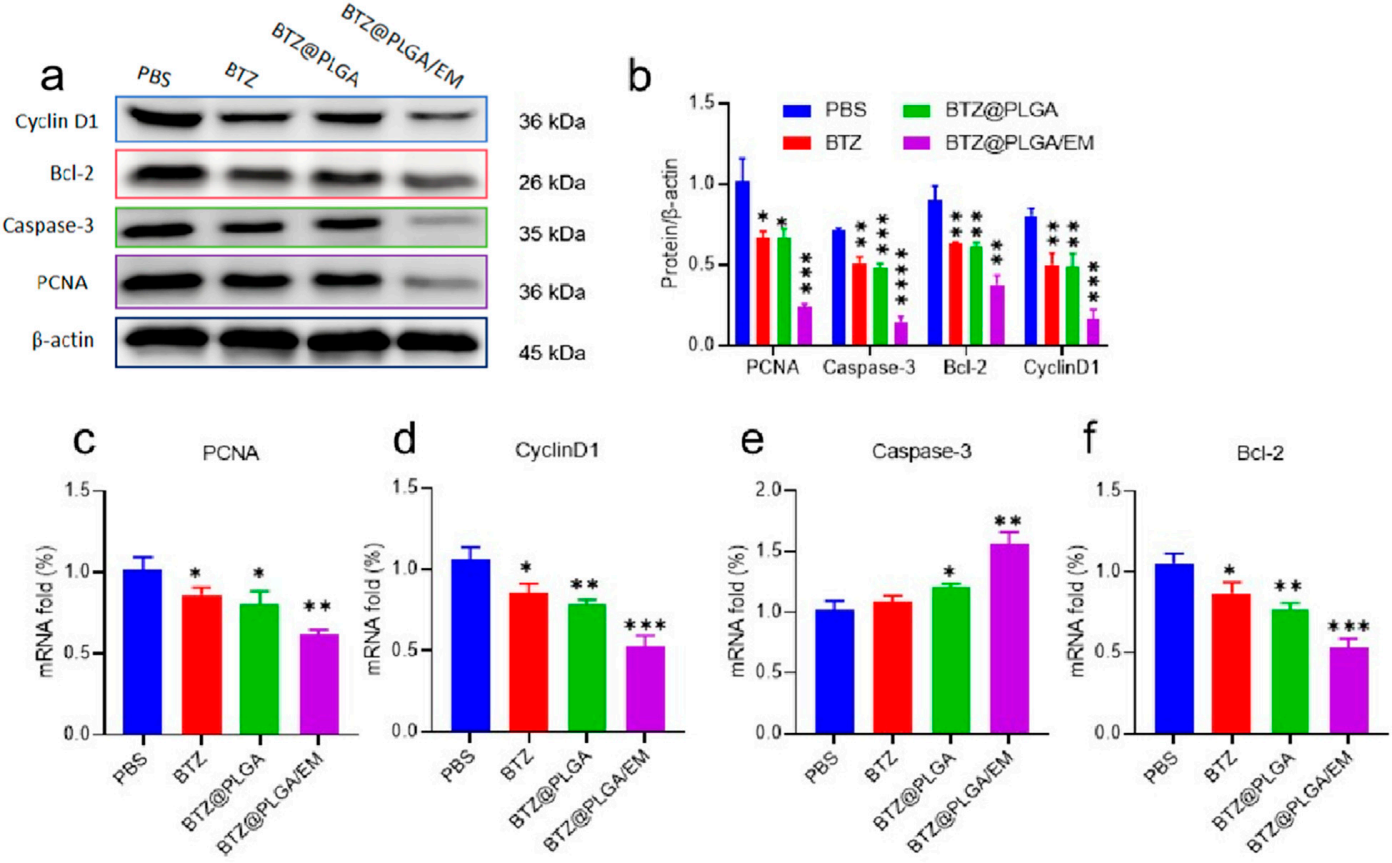
Expression of apoptosis-related proteins. **(a)** Western blot was used to detect the activity of cell cycle regulatory proteins Cyclin D1 and PCNA, anti-apoptotic proteins Bcl-2 and apoptotic proteins Caspase-3. **(b)** Quantitative analysis of Western blot results. **(c–f)** The mRNA expressions of PCNA, Cyclin D1, Caspase-3 and Bcl-2 were detected by PCR. Data are presented as mean ± SD (n = 3 independent experiments). Statistical significance was determined by one-way ANOVA followed by Tukey’s multiple comparison test. p < 0.05, p < 0.01, p < 0.001.

By competitively binding to the active site of proteasome, BTZ inhibits the degradation of related receptor proteins in cells, leading to the increase of intracellular stress, and then plays a role in promoting apoptosis. Cyclin D1 is one of the key cell cycle regulators, mainly through the interaction with CDK4 and/or CDK6 to regulate G1/S phase progression (Hentzen et al., 2020). Proliferating cell nuclear antigen (PCNA) is also a key protein in the cell cycle, which plays an important role in DNA replication and cell proliferation (Hentzen et al., 2020). The results of WB showed that the activity of Cyclin D1 and PCNA was decreased, and the results of PCR showed that the expression of Cyclin D1 and PCNA was decreased (Figures 4a–d). These results indicated that BTZ could inhibit the growth of MM cells. To confirm that BTZ induced apoptosis, cells incubated with BTZ, BTZ@PLGA and BTZ@PLGA/EM were subjected to Western blot and qPCR analysis for the expression of apoptosis-related proteins. Bcl-2 is an anti-apoptotic protein, and the downregulation of Bcl-2 promotes the occurrence of apoptosis (Zhang et al., 2024). Caspase-3 is an apoptosis-promoting protease with increased activity during early apoptosis and a marked decrease in activity during late apoptosis and necrosis (Wenzinger et al., 2018). The results of Western blot and PCR showed that the activities of Bcl-2 and Caspase-3 were decreased, it may be that Caspase-3 is activated leading to a decrease, while activated Caspase-3 is increased (Figures 4a,b). The results of PCR showed that the expression of Bcl-2 was decreased and the expression of Caspase-3 was increased (Figures 4e,f). These proved that the cells underwent apoptosis.

### *In vivo* targeting and bone marrow homing of BTZ@PLGA/EM nanovesicles

**FIGURE 5:**
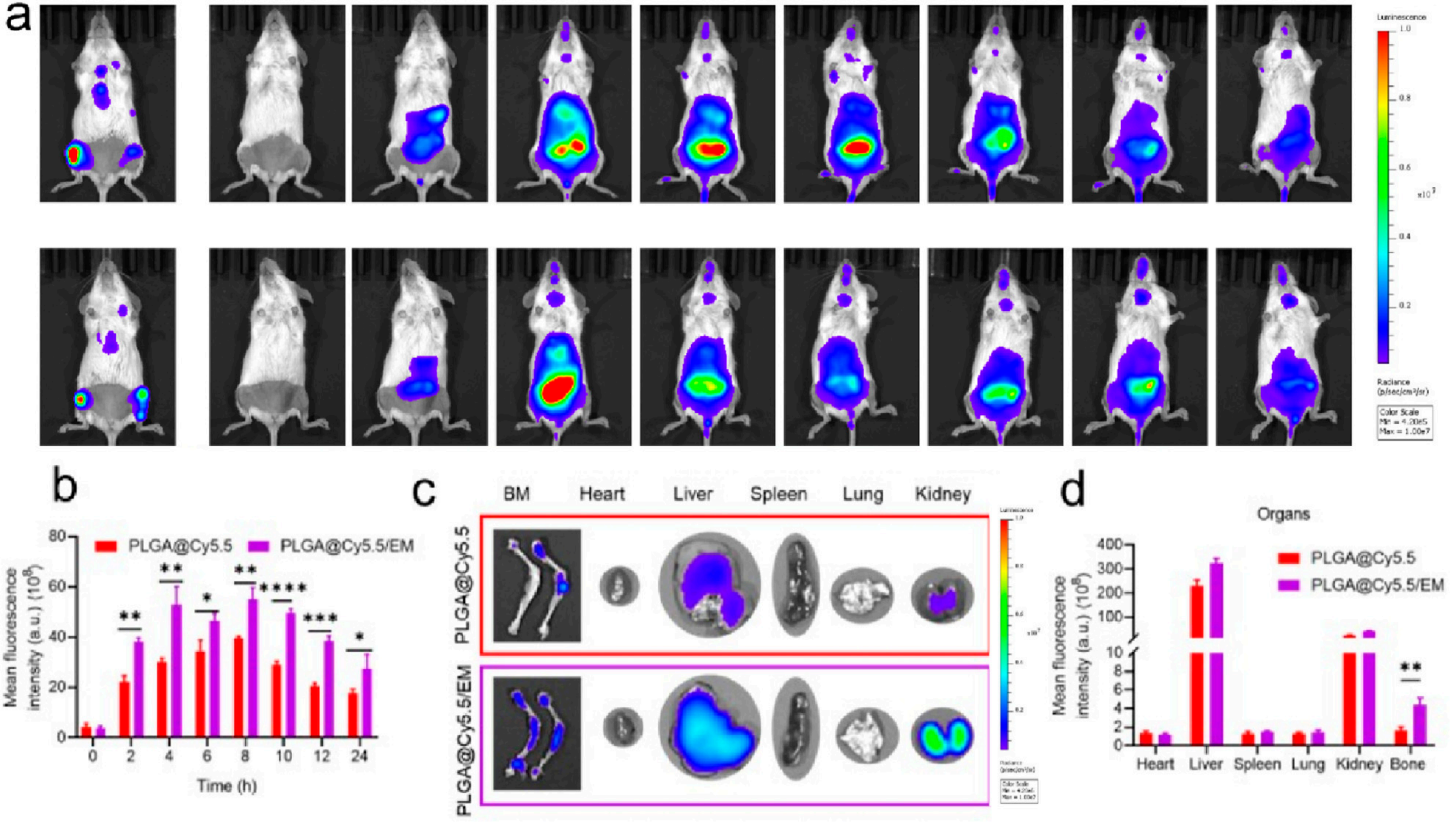
The targeting of engineered cell membranes to myeloma. **(a)** IVIS was used to observe the distribution of Cy5.5-labeled nanocapsules in Luc-labeled orthotopic myeloma mice at different time points. **(b)** Quantitative data on the fluorescence intensity of PLGA@Cy5.5 and PLGA@Cy5.5/EM in mice at different time points. **(c)** Images of fluorescence distribution of nanovesicles in isolated organs and femurs. **(d)** Fluorescence quantification of nanovesicles in isolated organs and femurs. Data are shown as mean ± SD (n = 3 mice). Statistical differences between groups were analyzed using two-tailed unpaired Student’s t-tests. p < 0.05, p < 0.01.

Luciferase transfected ARD cells (ARD-Luc) were injected intravenously into B-NDG mice to establish a primary myeloma model. After the model was successfully constructed, Cy5.5 labeled PLGA@Cy5.5 and PLGA@Cy5.5/EM nanovesicles with the same fluorescence intensity were injected through the tail vein. Then, a near-infrared fluorescence (IVIS) imaging system was used to observe the fluorescence enrichment in mice at different time points. As shown in Figure 5a, the fluorescence intensity at myeloma sites in mice injected with PLGA@Cy5.5/EM nanovesicles was higher than that injected with PLGA@Cy5.5 nanovesicles and enhanced with time, and the fluorescence gradually decreased from 8 h. This conclusion was also supported by the quantitative data of fluorescence (Figure 5b). After 24 h, the mice were dissected and the major organs and femoral tissues were collected for *ex vivo* imaging to observe the fluorescence distribution. The fluorescence of PLGA@Cy5.5/EM in myeloma was significantly higher than that of PLGA@Cy5.5, the quantitative data were significantly different, indicating that the engineered cell membrane had the target tumor site ability and bone marrow homing ability (Figures 5c,d).

### *In vivo* therapeutic efficacy of BTZ@PLGA/EM against multiple myeloma

**FIGURE 6:**
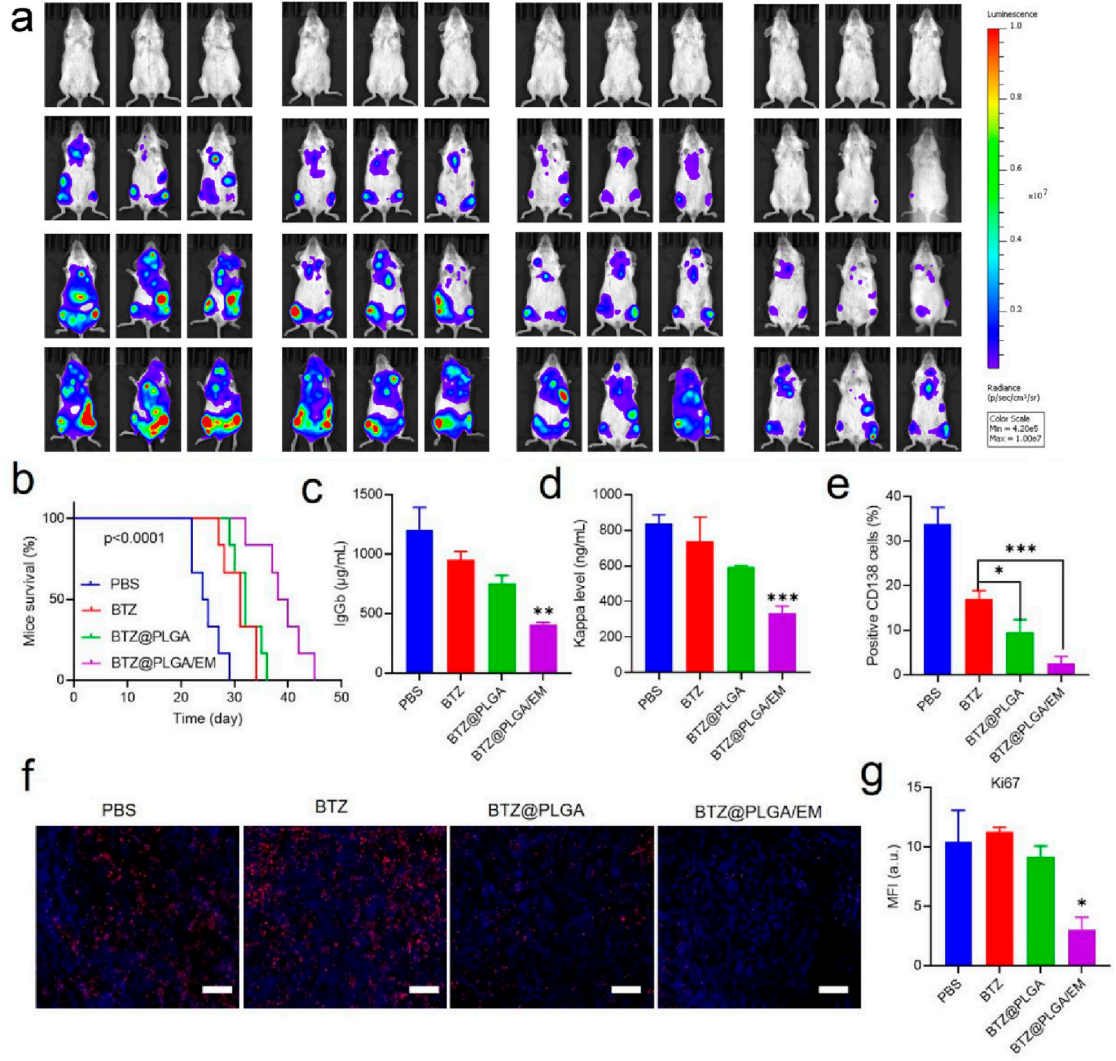
The therapeutic effect of BTZ@PLGA/EM on myeloma *in vivo*. **(a)** The Luc fluorescence of myeloma cells in mice treated with PBS, BTZ, BTZ@PLGA, BTZ@PLGA/EM for 1 week and 2 weeks was observed. **(b)** Survival curves of mice subjected to different treatments. **(c,d)** At the end of treatment, the levels of immunoglobulin IgGb and immunoglobulin light chain Kappa secreted by myeloma were detected in serum. **(e)** Myeloma cells were labeled with CD138 to detect the number of myeloma cells in mice after the end of treatment. **(f)** Ki67 staining was used to evaluate the proliferation activity of myeloma cells. **(g)** Fluorescence quantification of Ki67 staining. Data are expressed as mean ± SD (n = 5 mice per group). Survival curves were analyzed using the Kaplan-Meier method and compared by the log-rank (Mantel–Cox) test. Other comparisons were evaluated by one-way ANOVA followed by Tukey’s test. p < 0.05, p < 0.01, p < 0.001, ns = not significant.

To evaluate the anti-MM effect of BTZ@PLGA/EM *in vivo*, B-NDG mice were injected with 1 × 10^6^ ARD-Luc cells via the tail vein to establish an orthotopic MM model. After 10 days, the MM model was successfully established observed by the IVIS imaging system, the mice were randomly divided into 4 groups and injected with PBS, free BTZ, BTZ@PLGA nanovesicles, BTZ@PLGA/EM nanovesicles (0.4 mg/kg BTZ), respectively for once every 3 days for a total of three doses. After that, the progression of MM tumor cells was observed by IVIS spectral imaging system every 5 days, and the body weight of the mice was recorded every other day, while the survival curve of the mice was recorded. As shown in Figure 6a, after the injection of different components of the drug, the bioluminescence of the mice injected with PBS became stronger over time, indicating that the growth of MM tumors was not inhibited. The MM growth rate of the mice injected with free BTZ was slower than that of the mice injected with PBS, but tumor growth was still not inhibited. However, the fluorescence intensity of MM in the mice injected with BTZ@PLGA was significantly lower than that in the PBS and BTZ groups at the first week, and the growth of MM was inhibited over time. Surprisingly, no significant tumor cells were observed in mice injected with BTZ@PLGA/EM nanovesicles in the first week. A small residual MM signal appeared in week 2, indicating strong inhibition by BTZ@PLGA/EM. Quantitative analysis of the fluorescence of the bioluminescence also demonstrated the anti-MM tumor effect of BTZ@PLGA/EM (Supplementary Figure S5). During the treatment period, mice treated with BTZ@PLGA/EM did not show significant changes in body weight, whereas mice in the other groups lost weight, which may be related to the inhibition of tumor growth by BTZ@PLGA/EM (Supplementary Figure S6). This treatment strategy also significantly prolonged the survival of mice, with mice injected with PBS surviving for only 29 days compared to 45 days in mice injected with BTZ@PLGA/EM nanovesicles (Figure 6B).

### Reduction of myeloma burden and serum immunoglobulins by BTZ@PLGA/EM

Progressive myeloma (MM) is characterized by malignant plasma cell proliferation, and neoplastic clones often secrete intact immunoglobulin (Ig) or fragments of immunoglobulin (Ig), such as light chains. Therefore, after the treatment, the levels of immunoglobulin IgGb, and light chain Kappa in the serum of mice were detected. After BTZ@PLGA/EM treatment, serum levels decreased, indicating therapeutic benefit; these nanovesicles have a good effect on MM (Figures 6c,d). At the end of the treatment, the femur tissue of the mice in each group was collected and bone marrow single cell suspension was prepared. The bone marrow cells were identified by PE anti-human CD138 and PE-Cy7 anti-human CD319 antibodies. The content of MM cells in the bone marrow was assessed by screening the cells by flow cytometry. As shown in Figure 6e and Supplementary Figure S7, treatment with BTZ resulted in a small reduction in MM cells, a significant reduction in MM cells after BTZ@PLGA treatment, and very few MM cells after BTZ@PLGA/EM treatment, which was significantly higher than that in the BTZ@PLGA treatment group. Meanwhile, collected bone marrow was analyzed by HandE and Ki67 staining. BTZ and BTZ@PLGA caused limited apoptosis, whereas BTZ@PLGA/EM caused extensive apoptosis (Figure 6f). This is also demonstrated by the fluorescence quantitative data of the pictures (Figure 6g).

### *In vivo* biosafety evaluation of BTZ@PLGA/EM nanovesicles

**FIGURE 7:**
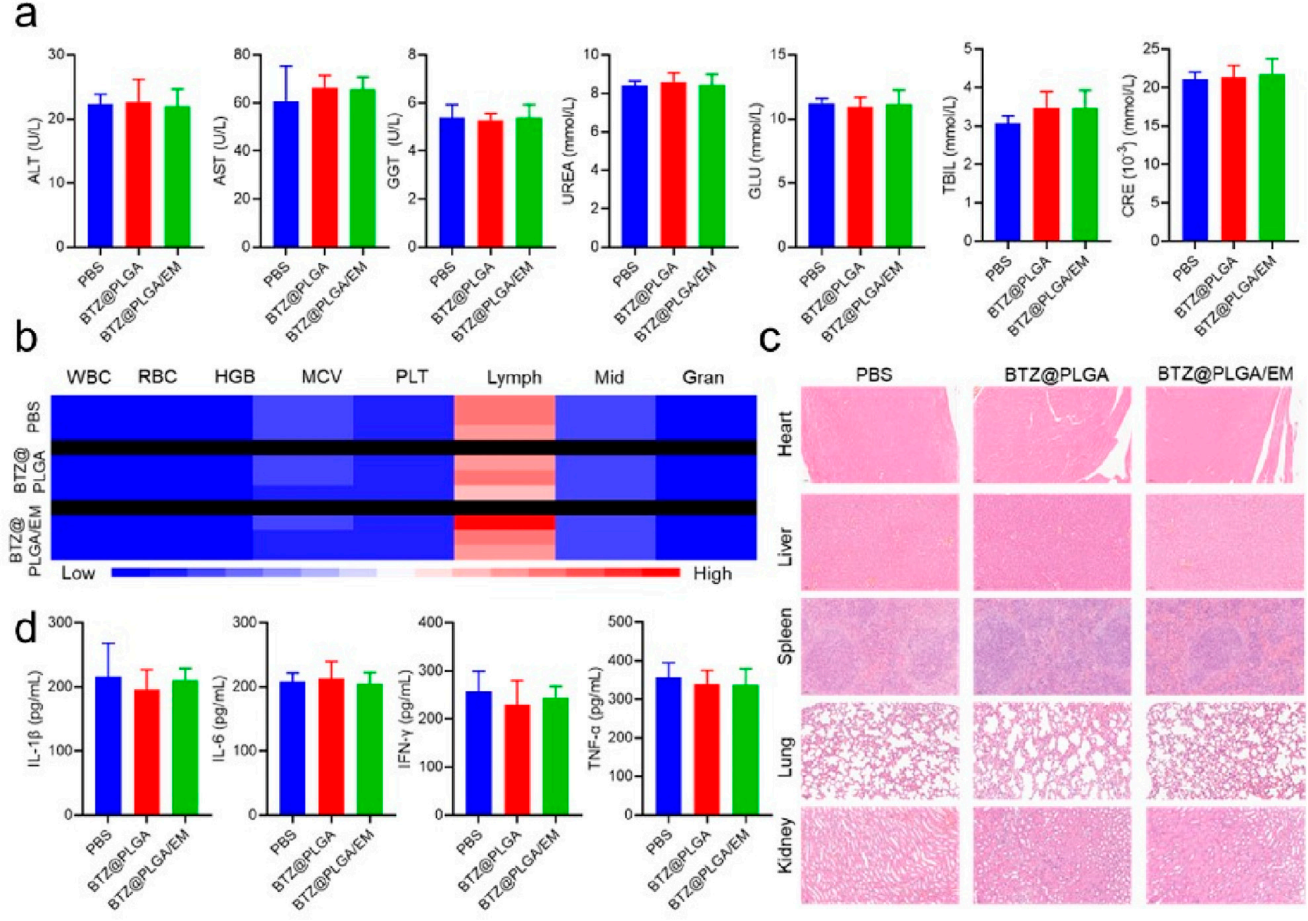
Biosafety evaluation after treatment *in vivo*. **(a)** Biochemical indicators in the serum of mice from different treatment groups. **(b)** The number of blood cells in the blood of mice from different treatment groups. **(c)** HandE staining of major organs of mice from different treatment groups. **(d)** The levels of inflammatory cytokines in the serum of mice in different treatment groups. Quantitative data are shown as mean ± SD (n = 3–5 mice). Statistical significance was assessed using one-way ANOVA with Tukey’s *post hoc* test. ns = not significant.

After the treatment, to demonstrate the biosafety of the nanovesicles, blood was collected from mice in each group for biochemical analysis and hemocyte analysis. The levels of alanine aminotransferase (ALT), Aspartate aminotransferase (AST), γ-glutamyl transpeptidase (GGT), UREA, glucose (GLU), total bilirubin (TBIL) and creatinine (CRE) did not change significantly in the BZT@PLGA and BZT@PLGA/EM treated groups compared with the PBS group (Figure 7a). Similarly, there were no abnormalities in blood cells such as white blood cells (WBC), red blood cells (RBC), hemoglobin (HGB), mean corpuscular volume (MCV), platelets (PLT), lymphocytes (Lymph), intermediate cells (Mid), and neutrophils (Gran) in the treated group compared with the PBS group (Figure 7b). HandE staining analysis was performed on the major organs of the mice after treatment in each group to determine whether there were abnormalities in each organ. As can be observed in Figure 7c, H&E staining showed that there were no abnormalities in the heart, liver, spleen, lung, and kidney in the BTZ@PLGA and BTZ@PLGA/EM groups compared with the PBS group. Finally, serum IL-1β, IL-6, IFN-γ, and TNF-α did not differ from PBS, indicating no immune stress from the treatment (Figure 7d). All these lines of evidence demonstrate *in vivo* biosafety.

## Discussion

The development of targeted nanomedicines that can efficiently penetrate the bone marrow microenvironment and selectively eliminate malignant plasma cells remains a major therapeutic challenge in multiple myeloma (MM). In this study, we designed and validated a sequentially dual-targeting biomimetic nanoplatform, BTZ@PLGA/EM, which synergistically combines bone-specific anchoring via alendronate modification with homotypic tumor targeting through MM cell membrane coating. This system significantly enhances the delivery efficiency and anti-myeloma efficacy of bortezomib (BTZ), while reducing its systemic toxicity.

One of the key innovations of our platform is its ability to achieve spatiotemporally controlled delivery through a “bone-tumor” sequential targeting mechanism. The initial binding to hydroxyapatite in the bone microenvironment enables preferential accumulation within myelomatous lesions, an advantage particularly critical in hematologic malignancies where the EPR effect—commonly exploited in solid tumors—is largely absent. Subsequently, the preserved adhesion molecules (e.g., CD44 and CD147) on the engineered membrane facilitate specific recognition and uptake by MM cells, thereby enhancing intracellular drug delivery and minimizing off-target effects. This two-step targeting strategy represents a significant advancement over conventional single-ligand or passive accumulation-based approaches, which often suffer from limited specificity and penetration in the complex bone marrow niche.

Our *in vitro* and *in vivo* results consistently demonstrated the superior targeting and therapeutic performance of BTZ@PLGA/EM. Enhanced cellular uptake and targeted accumulation were confirmed via fluorescence imaging and flow cytometry, while potent proteasome inhibition and apoptosis induction were validated through Western blotting, PCR, and apoptosis assays. Notably, the nanovesicles significantly extended the survival of MM-bearing mice, with all animals in the treatment group surviving until day 45, compared to uniform mortality by day 29 in the PBS control group. This pronounced therapeutic benefit was achieved without inducing significant systemic toxicity, as evidenced by unchanged biochemical, hematological, and inflammatory parameters, along with normal histology in major organs.

Importantly, the reduction in serum immunoglobulin levels and MM cell counts in the bone marrow further confirms the disease-modifying potential of our platform. The decreased expression of proliferation markers (PCNA, Cyclin D1) and activation of apoptotic pathways (Caspase-3, Bcl-2 downregulation) provide mechanistic insights into BTZ-induced cell death, underscoring the biological relevance of the targeted delivery strategy.

By integrating “bone homing” with “tumor recognition,” we have established a versatile targeting strategy that holds promise for extension to other blood cancers involving bone marrow infiltration. Future work will focus on evaluating this platform in clinically relevant models, including patient-derived xenografts and drug-resistant MM subtypes, to further validate its therapeutic potential. Moreover, the modular nature of this system allows for the incorporation of other therapeutic agents or imaging probes, supporting its development into a theranostic platform. Long-term toxicity studies, immune response monitoring, and scalable production represent critical steps toward clinical translation. Ultimately, this study presents a promising nanotherapeutic strategy for MM and suggests a route to treat disseminated malignancies in protective niches.

## Limitations of the study

While this study demonstrates the promising efficacy and safety of the sequentially targeted biomimetic nanoplatform BTZ@PLGA/EM, several limitations should be acknowledged. First, the research utilized immunodeficient B-NDG mouse models, which do not fully recapitulate the human immune microenvironment or potential immune responses to repeated administration of nanovesicles. Future studies in immunocompetent models or humanized mice would provide deeper insight into its biocompatibility and translational relevance. Second, although the membrane coating strategy showed enhanced homologous targeting, the efficiency of membrane fusion and functional protein orientation during extrusion remains challenging to quantitatively control, which may contribute to batch-to-batch variability. Furthermore, the long-term biological fate and potential accumulation of repeated doses of PLGA and engineered membranes were not thoroughly evaluated. Finally, while the reduction in systemic toxicity is encouraging, detailed neurotoxicity assessments—particularly important for BTZ-based therapies—were not included; such evaluations would be critical in future preclinical and clinical developments.

## Conclusion

In summary, we have successfully developed a sequentially dual-targeted nanovesicle system by leveraging the innate homologous targeting capability of tumor cell membranes and the bone-specific affinity of bisphosphonates for precise delivery of bortezomib (BTZ) to multiple myeloma (MM) cells within the bone marrow microenvironment. The biomimetic platform, BTZ@PLGA/EM, employs an active hierarchical targeting mechanism described as “bone-first, tumor-second,” which not only enhances drug accumulation at the disease site but also markedly reduces off-target toxicity. Accordingly, it addresses key limitations of conventional BTZ therapy, including short half-life, neurotoxicity, and inadequate bone-marrow penetration. The core advantage of this strategy lies in its ability to overcome the constraints of single-target approaches, enabling multi-level active navigation within the complex bone marrow milieu and offering a more precise delivery alternative for hematologic malignancies that lack the EPR effect.

## Data Availability

The original contributions presented in the study are included in the article/Supplementary Material, further inquiries can be directed to the corresponding author.
