# Supplementary material for: Sequential dual-targeting biomimetic nanovesicles for bone marrow–specific delivery of bortezomib in multiple myeloma
Source: Front Bioeng Biotechnol. 2025 Nov 17;13:1714613. doi: 10.3389/fbioe.2025.1714613 (PMC12665660; doi:10.3389/fbioe.2025.1714613)
Supplement: Supplementary file 1 [file Supplementaryfile1.docx]

*Supporting Information (SI)*

**Sequential Dual-Targeting Biomimetic Nanovesicles for Bone Marrow–Specific Delivery of Bortezomib in Multiple Myeloma**

**1. Experimental Section**

**1.1 BTZ@PLGA Nanoparticle preparation**

PLGA nanoparticles were prepared by oil-in-water (O/W) emulsification solvent evaporation method. 50mg PLGA dissolved in 4mL acetone was used as the organic phase. Under stirring, the above organic phase was gradually added to the 10mL aqueous phase. After the two phase solutions were mixed evenly, the organic phase solvent gradually evaporated and the substances in the solution gradually solidified to form nanoparticles. The obtained PLGA nanoparticles were placed in dialysis bags with a molecular weight interception of 8 to 12kDa, dialyzed in PBS solution for 48h, and stored at 4 ^o^C until later use. The acetone solution of PLGA (12.5mg/mL) was added drop by drop to the 0.5mg/mL aqueous solution of BTZ, and the agitation was continued until all drops were finished. After the organic phase was completely volatilized, BTZ-loaded nanoparticles (BTZ@PLGA) were obtained.

**1.2 Synthesize DSPE-PEG-Ald**

DSPE-PEG2000-COOH, EDC and NHS were mixed in DMSO solution at a mass ratio of 1:1:1.5 at 37 ° C for 30min to obtain DSPE-PEG2000-NHS. Subsequently, DSPE-PEG2000-NHS and Ald were reacted in DMSO solution at a molar ratio of 1:1 at room temperature for 6h. After removal of DMSO solution by rotary distillation and concentration, ice diethyl ether was added, and the product was placed at -20 ^o^C for 1h before filtration.

**1.3 MM tumor cell membrane extraction**

MM tumor cell membranes were extracted from ARD cells using membrane protein and plasma protein extraction kits. 6×10^8^ ARD cells were collected in 30 mL membrane protein extraction Reagent A (containing 100 mM PMSF, 300 μL). After 15 minutes in an ice water bath, the cells were repeatedly frozen and thawed three times. Subsequently, the cell suspension was centrifuged for 10 minutes (700 g, ^o^C) to collect the supernatant, and subsequently centrifuged again to collect the precipitate (14000 rpm, 4 ^o^C). 30 min at 4 ^o^C). After the total protein concentration was determined using the BCA kit, the samples were lyophilized and stored at -80 ^o^C.

**1.4 Preparation of BTZ@PLGA/EM**

500μL of freshly prepared BTZ@PLGA nanoparticles aqueous solution was mixed uniformly with 500 μl tumor cell membrane and 500 μl LDSPE-PEG-Ald, and extruded several times by Avanti micro extruder to pass through 200nm polycarbonate porous membrane. Subsequently, uniform-sized BTZ@PLGA/EM Nanoparticles were obtained.

**1.5 Release behavior of BTZ**

To assess the kinetics of bortezomib release from BTZ@PLGA/EM Nanoparticles, 1mg /mL BTZ@PLGA/EM was placed into a dialysis bag and incubated in PBS solution at 37°C. At the indicated time points, the nanovesicles were collected from the dialysis bag and dissolved in organic solvent, The content of BTZ was analyzed by liquid chromatography (HPLC).

**1.6 Characterization of nanovesicles**

The fresh BTZ@PLGA/EM Nanoparticles were diluted to the appropriate concentration with ultra-pure water and mixed evenly. The hydrated particle size, potential distribution and PDI value of the nanovesicles were measured by laser nanoparticle size analyzer. In addition, transmission electron microscopy (TEM) and scanning electron microscopy (SEM) were used to observe the morphology of BTZ@PLGA/EM nanocapsules. The successful synthesis of DSPE-PEG-Ald was characterized by Fourier transform infrared spectroscopy. SDS-PAGE gel electrophoresis was used to verify MM cell membrane specific protein markers on the surface of nanocapsules.

**1.7 In vitro cytotoxicity**

MM tumor cells (ARD) and normal cells (COS-7) were seeded at 106/ well in 96-well plates and treated with different concentrations of different BTZ preparations, including free bortezomib, blank PLGA nanoparticles, BTZ@PLGA nanoparticles, BTZ@PLGA/EM nanocarries. After incubation for 24 h and 48 h, cell viability was measured by adding CCK-8 and stored for 1-4 h.

**1.8 Specific tumor targeting**

ARD, COS-7 cells were seeded in confocal microscopy dishes. After 24 h of incubation, the medium was replaced with serum-free medium and the same concentration of Dio (green fluorescence) fluorescently labeled BTZ@PLGA/EM nanovesicles was added. After 4 h, the cells were washed three times with PBS and fixed with paraformaldehyde solution. Finally, the co-localization of the nanovesicles

and the cell membrane was studied by Hoechst staining, DiD staining and confocal microscopy (CLSM).

**1.9 Apoptosis and necrosis of MM cells**

ARD cells were seeded in 6-well plates at a density of 4×105 cells/well, and then treated with free bortezomib, blank PLGA nanoparticles, BTZ@PLGA nanoparticles, BTZ@PLGA/EM nanocapsules (BTZ concentration was 4ng/mL). After 12 hours, the cells in the Wells were collected. Cell apoptosis and necrosis were detected by PI and Annexin V staining and flow cytometry.

**1.10 Expression of BTZ-related proteins**

ARD cells were seeded in 6-well plates at a density of 4×105 cells/well, and then treated with free bortezomib, blank PLGA nanoparticles, BTZ@PLGA nanoparticles, BTZ@PLGA/EM nanocapsules (BTZ concentration was 4ng/mL). After 12 hours, the cells in the Wells were collected. The expressions of PCNA, cyclin D1, Bcl-2 and Caspase-3 were detected by polymerase chain reaction (PCR) and Western Blot (WB), respectively.

**1.11 Establishment of orthotopic mouse myeloma model**

The human MM cell line (ARD-luc) transfected with 1×106 luciferase was injected intravenously into 6-week-old B-NDG mice. After 10 days, tumor cells were localized in the bone marrow for proliferation, and successful establishment of myeloma was determined with the IVIS spectral imaging system.

**1.12 Bone marrow homing and targeting ability in vivo**

1×10^6^ ARD-luc cells were injected intravenously into 6-week-old B-NDG mice. After 10 days, the IVIS imaging system observed the colonization of the tumor cells in the bone marrow. Dir-labeled DiR@PLGA nanoparticles, DiR@PLGA/EM nanocapsules, and free DiR were administered intravenously. The location of tumors and the biodistribution of nanocapsules were observed by IVIS imaging system at specified time points (1 h, 2 h, 4 h, 8h, 12 h, 24 h, 48h). The main tissues and organs (heart, liver, spleen, lung, kidney) and bone tissues were dissected for ex vivo organ imaging at 48h.

**1.13 Evaluation of anti-tumor effect of nanovesicles**

1×10^6^ ARD-luc cells were injected intravenously into 6-week-old B-NDG mice. After 10 days, mice were randomly divided into five groups. PBS, free BTZ, blank PLGA nanoparticles, BTZ@PLGA nanoparticles, and BTZ@PLGA/EM nanocapsules (0.4 mg/kg BTZ) were injected intravenously once every three days for a total of three doses. After that, the progression of MM tumor cells was observed by IVIS spectral imaging system every 5 days, and the body weight of the mice was recorded every other day, while the survival curve of the mice was recorded.

**1.14 Flow cytometry analysis and pathological section analysis of MM cells in bone marrow**

After different drug treatments, 3 mice in each group were sacrificed, femur tissue was collected and bone marrow single cell suspension was prepared. Bone marrow cells were identified by PE anti-human CD138 and PE-Cy7 anti-human CD319 antibodies. Finally, cells were screened by flow cytometry to assess the MM cell content in the bone marrow. Meanwhile, the collected bone tissues were analyzed for apoptosis of tumor cells by H&E staining and dUTP immunofluorescence staining.

**1.15 Biological safety**

Three healthy mice were randomly selected from each group after different drug treatments, and peripheral blood and main tissues and organs were collected. Blood biochemistry, blood routine analysis, and H&E staining were used for pathological analysis of tissues and organs to study the biosafety of nanovesicles in vivo.

**1.16 Statistical Analyses**

All quantitative data are presented as mean ± standard deviation (SD) from at least three independent biological replicates. Statistical analyses were performed using GraphPad Prism 9.0 (GraphPad Software, USA). Comparisons between two groups were conducted using two-tailed unpaired Student’s t-tests, while multiple group comparisons were analyzed by one-way ANOVA followed by Tukey’s multiple comparison test. Survival data were evaluated using the Kaplan–Meier method and compared by the log-rank (Mantel–Cox) test. A p value < 0.05 was considered statistically significant. Significance levels are indicated as follows: p < 0.05, p < 0.01, p < 0.001, and p ≥ 0.05 (ns, not significant).

**2. Supplementary Figures**


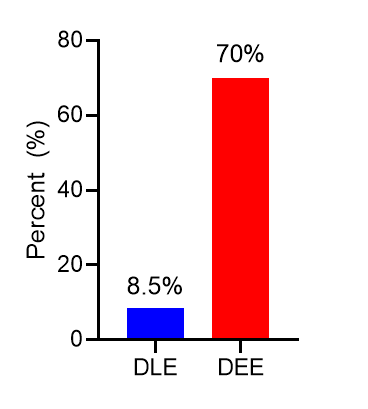


Figure S1. The amount of BTZ encapsulated in the nanocapsules. DLE: Drug loading efficiency; DEE: Drug encapsulation efficiency.


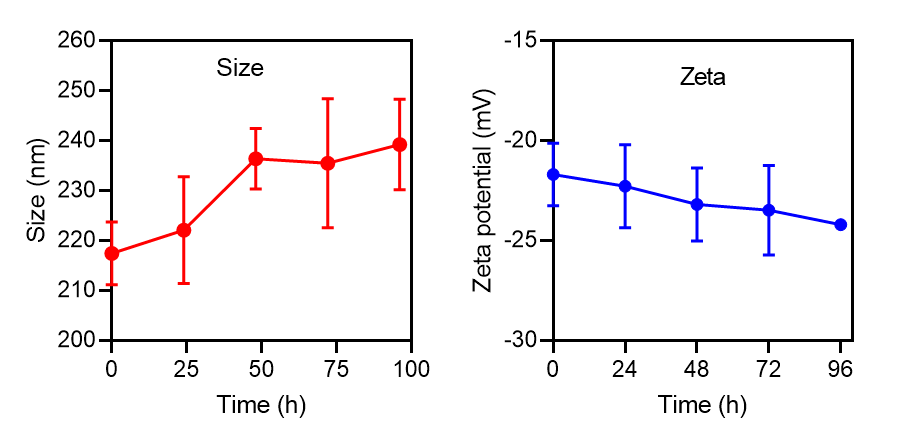


Figure S2. BTZ@PLGA particle size and zeta potential changes at different time points in PBS.

Figure S3. Expression of CD47 and CD147 on MM cells.


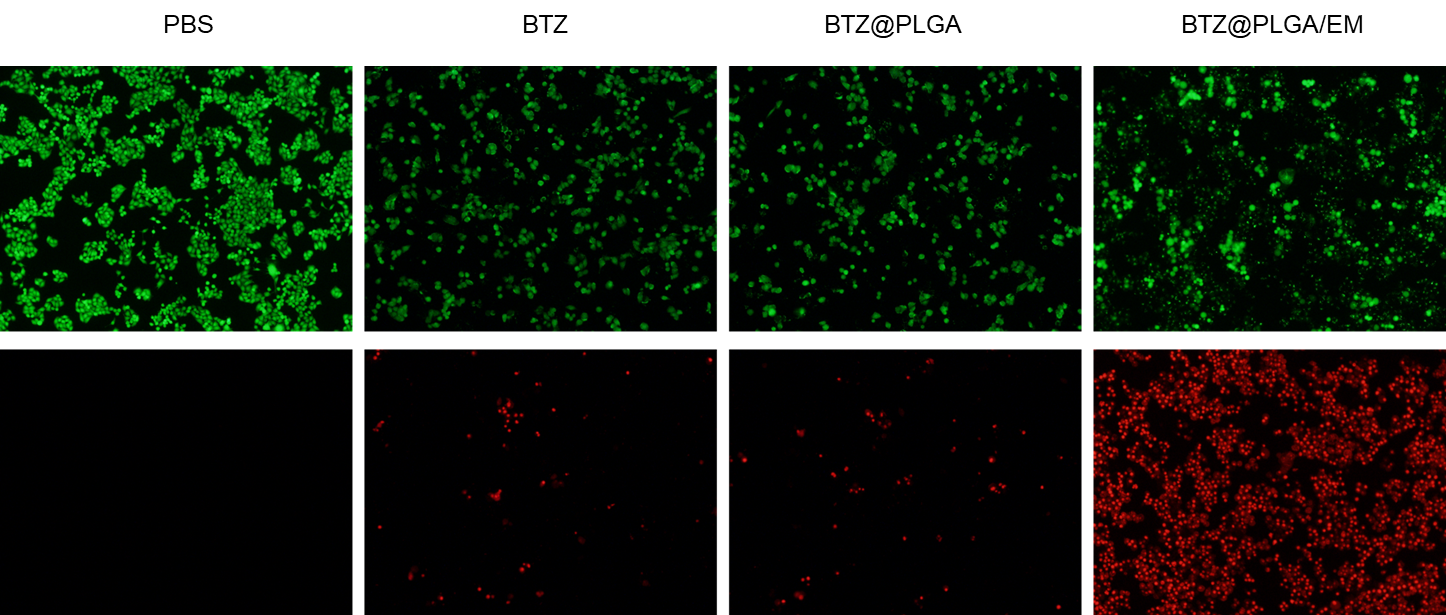


Figure S4. Live and dead staining of MM cells after co-incubation with PBS, BTZ, BTZ@PLGA, BTZ@PLGA/EM.


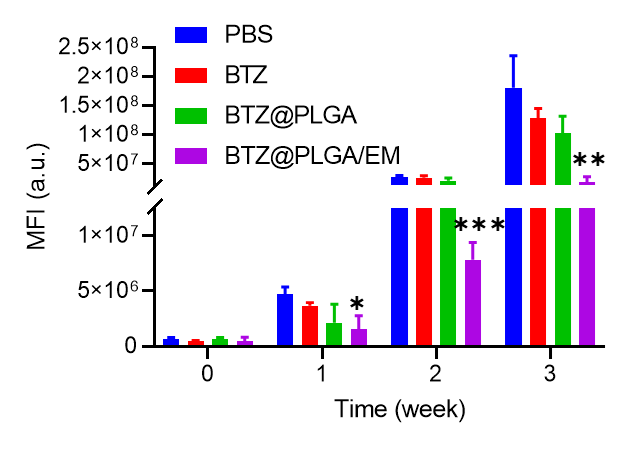


Figure S5. In vivo fluorescence quantitative data for anti-MM therapy.


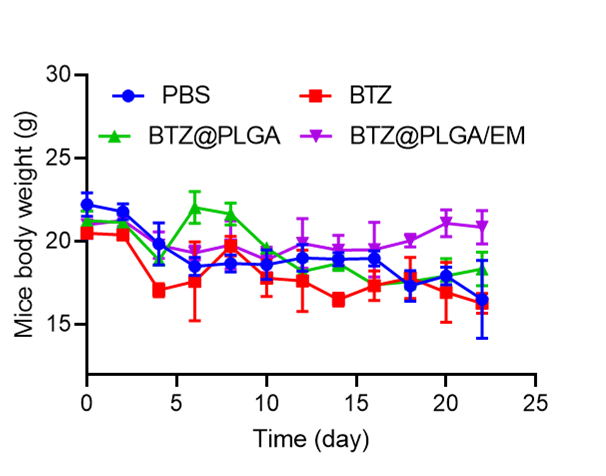


Figure S6. Weight change curves of mice in each group during treatment.


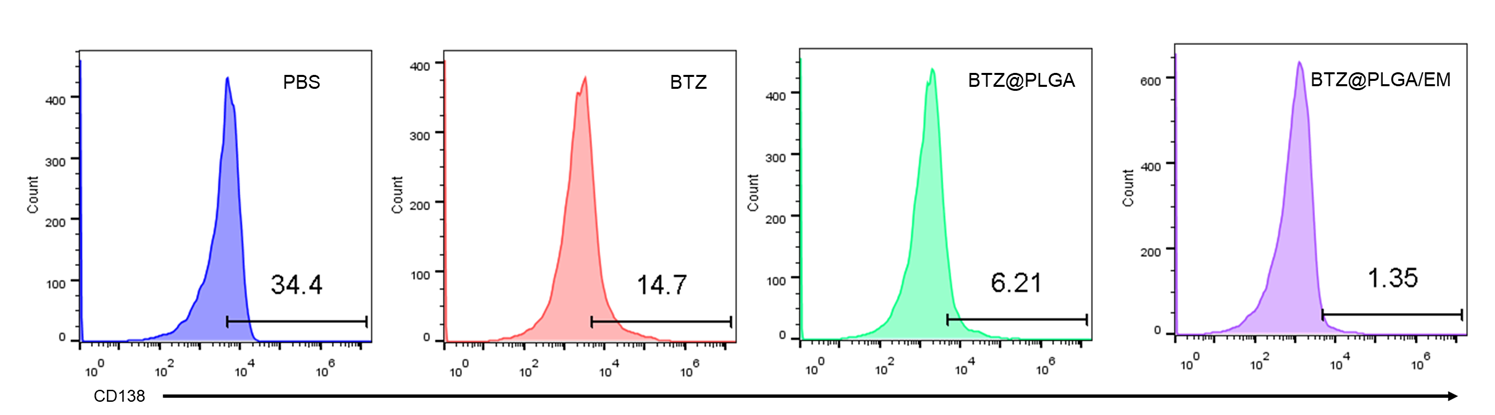


Figure S7. Quantification of MM cells by flow cytometry after treatment.
