# Supplementary material for: Sequential dual-targeting biomimetic nanovesicles for bone marrow–specific delivery of bortezomib in multiple myeloma
Source: Front Bioeng Biotechnol. 2025 Nov 17;13:1714613. doi: 10.3389/fbioe.2025.1714613 (PMC12665660; doi:10.3389/fbioe.2025.1714613)
Supplement: Supplementary file 2 [file Presentation1.pptx]

## Slide 1
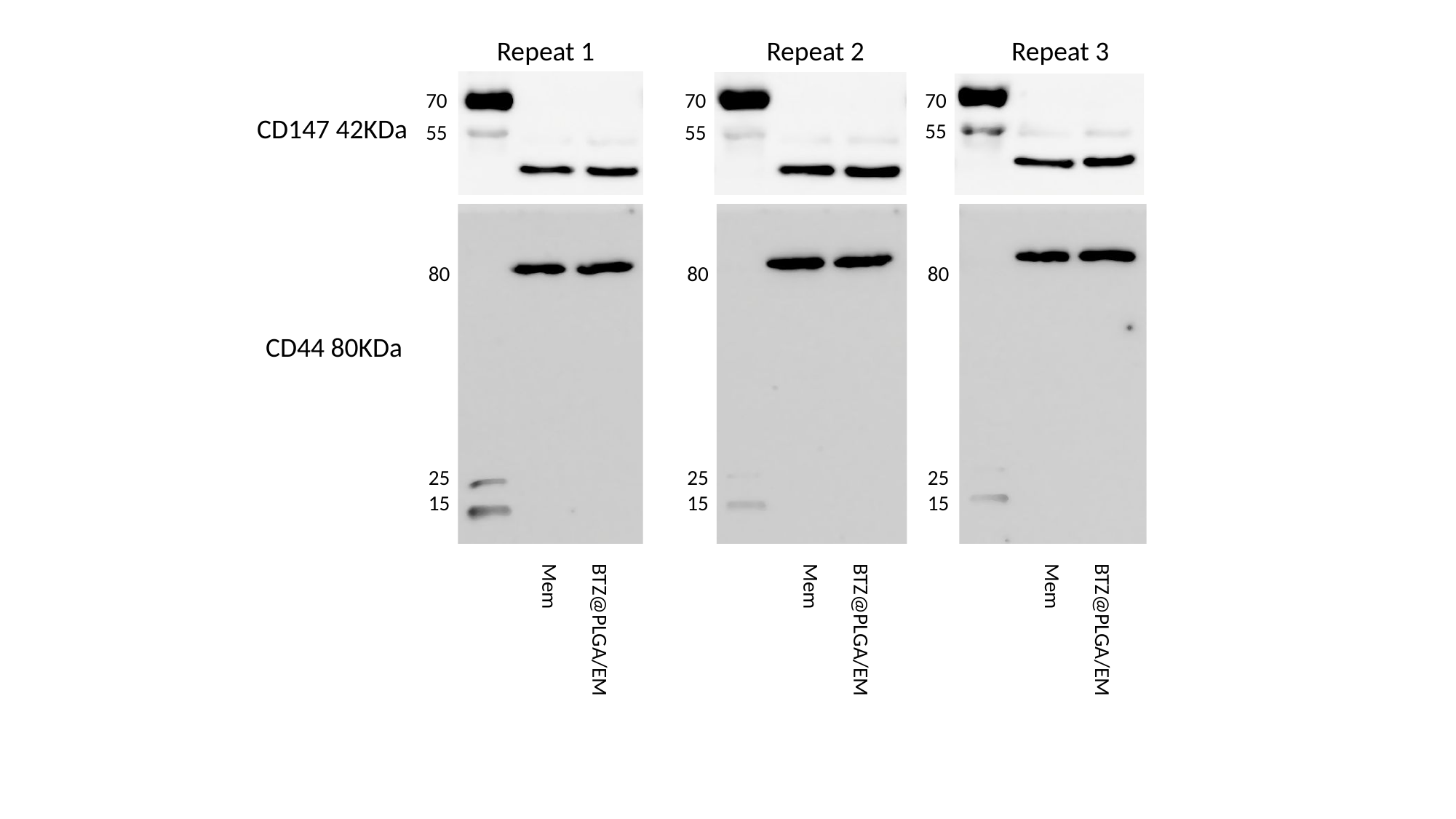

Repeat 1
Repeat 2
55
Repeat 3
55
70
70
70
CD147 42KDa
55
80
25
15
80
25
15
80
25
15
CD44 80KDa
Mem
BTZ@PLGA/EM
Mem
BTZ@PLGA/EM
Mem
BTZ@PLGA/EM

## Slide 2
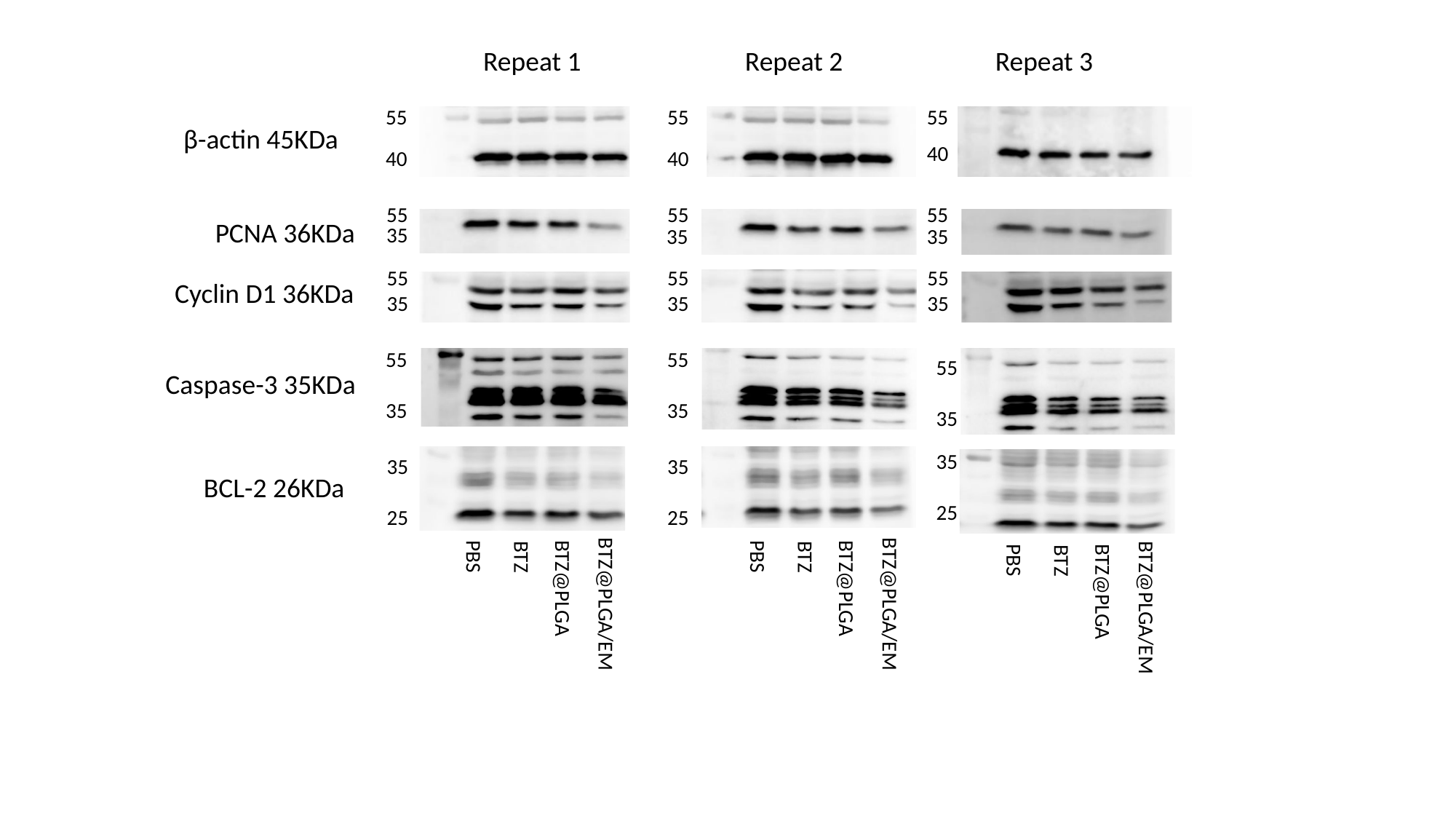

Repeat 1
Repeat 2
Repeat 3
55
55
55
β-actin 45KDa
40
40
40
55
55
55
PCNA 36KDa
35
35
35
55
35
55
35
55
35
Cyclin D1 36KDa
55
35
55
35
55
35
Caspase-3 35KDa
35
25
35
25
35
25
BCL-2 26KDa
BTZ
PBS
BTZ@PLGA
BTZ@PLGA/EM
BTZ
PBS
BTZ@PLGA
BTZ@PLGA/EM
BTZ
PBS
BTZ@PLGA
BTZ@PLGA/EM
